# Modulation of the Rat Hepatic Cytochrome P4501A Subfamily Using Biotin Supplementation

**DOI:** 10.1155/2013/627907

**Published:** 2013-07-28

**Authors:** M. D. Ronquillo-Sánchez, R. Camacho-Carranza, C. Fernandez-Mejia, S. Hernández-Ojeda, M. Elinos-Baez, J. J. Espinosa-Aguirre

**Affiliations:** Instituto de Investigaciones Biomédicas, Universidad Nacional Autónoma de México, Apdo. Postal 70228, 04510 México, DF, Mexico

## Abstract

Studies have found that biotin favors glucose and lipid metabolism, and medications containing biotin have been developed. Despite the use of biotin as a pharmacological agent, few studies have addressed toxicity aspects including the possible interaction with cytochrome P450 enzyme family. This study analyzed the effects of pharmacological doses of biotin on the expression and activity of the cytochrome P4501A subfamily involved in the metabolism of xenobiotics. Wistar rats were treated daily with biotin (2 mg/kg, i.p.), while the control groups were treated with saline. All of the rats were sacrificed by cervical dislocation after 1, 3, 5, or 7 days of treatment. CYP1A1 and CYP1A2 mRNAs were modified by biotin while enzyme activity and protein concentration were not affected. The lack of an effect of biotin on CYP1A activity was confirmed using other experimental strategies, including (i) cotreatment of the animals with biotin and a known CYP1A inducer; (ii) the addition of biotin to the reaction mixtures for the measurement of CYP1A1 and CYP1A2 activities; and (iii) the use of an S9 mixture that was prepared from control and biotin-treated rats to analyze the activation of benzo[a]pyrene (BaP) into mutagenic metabolites using the Ames test. The results suggest that biotin does not influence the CYP1A-mediated metabolism of xenobiotics.

## 1. Introduction

The vitamin biotin acts as a covalently bound coenzyme of carboxylases. Unrelated to this role, biotin supplementation modifies gene expression [1–5] and displays a wide repertoire of effects on systemic processes [6]. DNA microarray studies and high-throughput immunoblotting studies have aided in the identification of thousands of genes whose expression levels are modified by biotin at both the transcriptional and the posttranscriptional levels [5, 7]. Biotin supplementation modifies the expression of critical genes that are involved in the regulation of carbohydrate and lipid metabolism [8–14]. In agreement with these findings, several observations have indicated that biotin supplementation improves glucose and triglyceride homeostasis [15–19], which has led to the development of commercially available medications containing pharmacologically relevant amounts of biotin (2 mg/day) in combination with chromium picolinate [20, 21]. 

Despite its use as a pharmacological agent, few studies have addressed the toxicity of biotin [22–25]. DNA microarray studies have provided evidence that biotin supplementation increases the levels of mRNA encoding cytochrome P450 (CYP)1B1 in human peripheral blood mononuclear cells *in vitro* [5]. In addition, the transcriptional activation of this gene was associated with the increased activity of CYP1B1 in human lymphoid cells and with the increased frequency of single-stranded DNA breaks [26]. 

CYP enzymes are a superfamily of hemoproteins that metabolize endogenous compounds and foreign substances (xenobiotics) in several organisms, including mammals [27]. The CYP1 family members are particularly important due to their capacity to “activate” a broad variety of procarcinogens and some drugs, leading to the formation of highly reactive metabolites that can react with macromolecules, including proteins, lipids, and nucleic acids [28, 29]. Additionally, CYP expression is further subject to chemical induction or inhibition that results in quantitative variations in metabolic activity. The modulation of CYP expression is implicated in at least two scenarios: (i) drug-drug interactions, leading to adverse health effects and (ii) inhibition of mutagenic/carcinogenic compound metabolism, leading to antimutagenic/anticarcinogenic effects. Therefore, the evaluation of the capacity of any new compound that is intended for human use to interact with the CYP enzymes that are involved in xenobiotic metabolism is mandatory.

Although biotin consumption is considered to be safe [22], studies evaluating its toxicity at high doses need to be carefully conducted, as biotin supplementation affects gene expression and physiological functions. In this study, we analyzed the effects of pharmacological doses of biotin on the expression and activity of members of the CYP1A subfamily that are involved in the metabolism of procarcinogens and some drugs.

## 2. Materials and Methods

### 2.1. Chemicals

Biotin, ethoxyresorufin (ER), methoxyresorufin (MR), resorufin, dimethyl sulfoxide (DMSO), benzo[a]pyrene (BaP), and NADPH were purchased from Sigma Chemical Co. (St. Louis, MO, USA). Rabbit polyclonal anti-rat CYP1A1 and rabbit polyclonal anti-rat CYP1A2 were both purchased from Chemicon International Inc. (Atlanta, GA, USA), Mouse polyclonal anti-rat GAPDH was obtained from Millipore (Billerica, MA, USA). Rabbit anti-mouse peroxidase and goat anti-rabbit peroxidase were obtained from Invitrogen Life Technologies (CA, USA). The chemicals that were used for electrophoresis and the nitrocellulose membranes were purchased from Bio-Rad Laboratories (Richmond, CA, USA). Trizol reagent, M-MLV reverse transcriptase, and oligo dT primers were purchased from Invitrogen Life Technologies (CA, USA). Taq Man universal PCR master mix, unlabeled PCR primers, and TaqMan MGB probes were purchased from Applied Biosystems (Foster City, CA, USA).

### 2.2. Animals

This study was approved by the Ethical Committee for Experimentation of the Biomedical Research Institute of the National Autonomous University of Mexico. Male Wistar rats (200–250 g body weight) were obtained from the animal facility at the Biomedical Research Institute of the National Autonomous University of Mexico and were handled according to the standard procedures that were established by the Ethical Committee for Experimentation at the same institute. Animals were maintained under a 12/12-hr light/dark cycle and were allowed to consume water and food *ad libitum* throughout the experimental periods, with the exception of the fasting period prior to sacrifice.

### 2.3. *In Vivo* Studies

#### 2.3.1. Animals Treated with Biotin

Thirty-two rats were divided into eight groups: four control groups and four experimental groups (each group contained four animals). The control groups were treated daily with saline (phosphate-buffered, i.p.), and the experimental groups were treated daily with biotin (2 mg/kg, i.p.) at doses that were equivalent to pharmacological doses of biotin. All of the rats were sacrificed by cervical dislocation after 1, 3, 5, or 7 days of treatment.

#### 2.3.2. Animals Treated with Biotin and Benzo[a]pyrene

We examined the effects of treatment with biotin in combination with BaP, a well-known CYP inducer, to examine the possible additive effects of these agents. Twenty rats were divided into four groups (five animals per group): control (saline), biotin treated (2 mg/kg, i.p.), BaP treated (15 mg/kg, i.p.), and biotin + BaP treated (2 mg/kg of biotin and 15 mg/kg of BaP). All of the rats were sacrificed by cervical dislocation after 24 hrs of treatment. Liver microsomes were prepared to measure the ethoxyresorufin O-deethylase (EROD) and methoxyresorufin *O*-demethylase (MROD) activities. 

#### 2.3.3. Preparation of the Liver S9 and Microsomal Fractions of Treated Animals

Liver S9 fractions were prepared according to the procedure described by Maron and Ames [30]. The rats were sacrificed by cervical dislocation, after which point their livers were rapidly removed, weighed, and washed in a 0.15 M KCl solution and minced. Each sample was separately homogenized in the same solution (3 mL/g liver). The homogenates were centrifuged at 9,000 ×g for 10 min, and the supernatants were stored at −80°C. A portion of this supernatant fraction was centrifuged at 105,000 ×g for 60 min aseptically at 4°C. The microsomal pellet was resuspended in a phosphate-buffered solution (67.5 mM K_2_HPO_4_ and 32.5 mM KH_2_PO_4_, pH = 7.4) and centrifuged again. Finally, the microsomes were stored in the same phosphate-buffered solution containing 1 mM dithiothreitol, 1 mM EDTA, and 20% glycerol, aliquoted into a series of labeled vials, and frozen at −80°C. The protein content of each sample was determined using the method outlined by Bradford [31].

#### 2.3.4. RNA Isolation and Quantitative Real-Time RT-PCR Expression Analysis of the Cytochrome P450 Enzymes

Total RNA was extracted from frozen liver tissues using Trizol reagent (Invitrogen Life Technologies, CA, USA). The RNA concentration was determined by measuring the optical density at 260 nm, and the purity was determined by calculating the OD_260_/OD_280_ absorption ratio (all ratios were ensured to be >1.8). RNA integrity was confirmed by electrophoresis on a 1% denaturing agarose gel. The chemicals that were used for reverse transcription were purchased from Invitrogen Life Technologies (CA, USA). The isolated RNA was reverse-transcribed using Moloney murine leukemia virus reverse transcriptase (M-MLV RT). Briefly, the RNA was denatured by heating it at 65° for 5 min, cooled on ice, and incubated in a reverse transcriptase reaction mixture. The standard mixture contained 1 *μ*g of total RNA, 40 units/*μ*L of RNaseOUT recombinant ribonuclease inhibitor, 10 mM concentrations of each of the dNTPs, 1 *μ*L of oligo (dT)_12–18_, 4 *μ*L of 5X first-strand buffer, 2 *μ*L of 0.1 M DTT, and 200 U of M-MLV reverse transcriptase in a total volume of 20 *μ*L. For reverse transcription, the tubes were incubated at 37° for 50 min, followed by rapid cooling.

PCR was performed using an ABI PRISM 7500 sequence detection system (Applied Biosystems (Foster City, CA, USA) using melting, annealing, and extension cycling conditions of 95° for 15 sec, 50° for 2 min, and 60° for 1 min. All amplifications were repeated for 40 cycles. Taq-Man Gene expression primers (Applied Biosystems, Foster City, CA, USA) were used to detect CYP1A1 (Rn 00487218_m1), CYP1A2 (Rn 00561082_m1), and GAPDH (Rn 99999916_s1). The quantitative expression levels of the genes were calculated based on the cycle threshold (CT) value of each sample at the linear part of the curve using the 2^−ΔΔCT^ relative quantification method [32]. All of the samples were assayed in triplicate. The values for each gene were normalized to the value of the housekeeping gene, GAPDH.

#### 2.3.5. Quantitative Real-Time RT-PCR Analyses of miR-27b, miR-122, and miR-328a Expression

To evaluate the concentrations of miR-27b, miR-122, and miR-328a, we performed quantitative real-time reverse transcription PCR assays in rat liver samples. The total RNA and miRNA fractions were isolated from the livers of control and biotin-supplemented rats using a small RNA isolation kit (Ambion, CA, USA). The miRNA expression levels were quantified using the mirVana qRT-PCR miRNA detection protocol (Ambion, CA, USA). The primers used were obtained from Applied Biosystems (Foster City, CA, USA) to detect miR-27b (rno-miR-27b* 464436_mat), miR-122 (rno-mir-122* 463893-mat), and miR-328a (rno-mir-328a* 462041-mat). Mature miRNA U87 was used as internal reference to normalize the RNA levels of the genes being studied. Each PCR reaction was performed in triplicate in a 20 *μ*L volume using TaqMan MicroRNA assays (Applied Biosystems) for 10 min at 95°C, followed by 40 cycles at 95°C for 15 s and 60°C for 60 s in an ABI PRISM 7500 Sequence Detection System (Applied Biosystems). The miRNA levels were quantified using the 2^−ΔΔCT^ relative quantification method [32] using the following formula: 2(CTmicroRNA-CTU87).

#### 2.3.6. CYP1A1 and CYP1A2 Activities

The formation of resorufin after *O*-dealkylation of 7-ethoxyresorufin and 7-methoxyresorufin, which are metabolic probes for the activity of CYP1A1 and CYP1A2, respectively, was measured spectrofluorometrically according to the procedure outlined by Burke et al. [33]. The catalytic activities were calculated from a standard curve of resorufin (5–500 pmol/mL). The excitation and emission wavelengths were set at 530 and 590 nm, respectively.

#### 2.3.7. Western Blot Analyses

Hepatic microsomes were used for the determination of CYP activity. Ten micrograms of the microsomal proteins from individual animals were separated using 7.5% SDS-PAGE and were transferred to 0.45 mm nitrocellulose sheets overnight [34]. The nitrocellulose membranes were blocked for 1 hr with 5% nonfat dry milk in phosphate-buffered saline at 4°C [35]. After a 10 min wash with PBS containing 0.3% Tween-20, the membranes were incubated with the corresponding anti-rat primary antibodies, anti-CYP1A1 (1 : 10000), and anti-CYP1A2 (1 : 10000), at room temperature for 1 hr. The membranes were then incubated with horseradish peroxidase-conjugated anti-rabbit or anti-mouse IgG secondary antibodies at room temperature for 1 hr (1 : 6400). To confirm equal loading in each lane, the protein levels of the CYP enzymes were normalized to the GAPDH protein levels. The proteins of interest were revealed using Luminol. Relative increases in band intensity over the controls for each CYP isoform were determined using 1D Kodak 3.6.3 v computer software.

### 2.4. *In Vitro* Studies

#### 2.4.1. Biotin-CYP Interactions

We determined both the EROD and MROD activities in the microsomes of rats that had been treated with phenobarbital (60 mg/kg, i.p., for the first three days and 30 mg/kg, i.p., on day four) and *β*-naphthoflavone (80 mg/kg, i.p., on day three) in the presence or absence of different concentrations of biotin to explore whether this vitamin could interfere with these enzyme activities. Buffer (50 mM Tris-HCl and 25 mM MgCl_2_, pH = 7.6), the substrate (dissolved in DMSO), and NADPH were incubated at 37°C for 3 min in a fluorometry cuvette. The enzymatic reactions were initiated by adding total microsomal proteins (200 *μ*g) at the same time that we added different concentrations of biotin (1, 2.5, 5, and 10 *μ*g/mL). The reactions were followed for 3 min, and the fluorescence was recorded every 15 s. The catalytic activities were calculated from a standard curve of resorufin (5–500 pmol/mL). The excitation and emission wavelengths were set at 530 and 590 nm, respectively.

#### 2.4.2. Ames Test

The *Salmonella* mutagenicity plate incorporation test was performed according to the method described by Maron and Ames [30]. Bacterial cultures were obtained by the inoculation of a liquid nutrient broth with the *S. typhimurium* strain TA98 and were incubated at 37°C with shaking overnight. We examined the liver S9 fractions from both control rats and rats that had been treated with biotin (2 mg/kg, i.p.) for 1, 3, 5, and 7 days. The S9 mixture consisted of the S9 fraction (0.1 mL/mL of S9), MgCl_2_ (8 mM), KCl (33 mM), glucose 6-phosphate (5 mM), and NADP^+^ (4 mM) in 50 mM phosphate buffer (pH = 7.4). The bacterial cultures (0.1 mL), BaP(1, 5, 10, and 20 *μ*g per plate), and the S9 mixture (0.5 mL) were mixed with soft agar and poured onto Petri dishes containing Vogel-Bonner minimal medium. The plates were incubated for 48–72 hrs, and the numbers of revertant colonies (His^+^) were recorded.

### 2.5. Statistical Analyses

All statistical analyses were performed using commercially available GraphPad Prism v 4.0 (La Jolla, CA, USA) software. The data were expressed as the means ± SEM. Statistical significance was assessed using Student's *t*-test, with the levels of significance set at *P* ≤ 0.05.

## 3. Results

### 3.1. Effects of Biotin on P450 mRNA Levels

**Figure 1:**
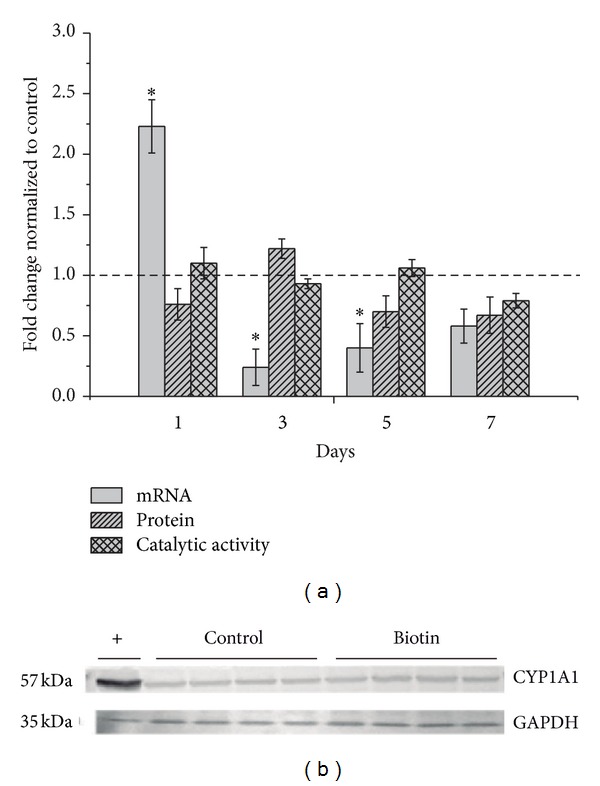
Relative CYP1A1 mRNA, protein, and catalytic activity levels after 1, 3, 5, and 7 days of biotin treatment (2 mg/kg, i.p.). The data are expressed as fold changes relative to the control group (dashed line). The data represent the means ± SEM of 4 rats per group from two independent experiments. *Significantly different from the controls (*P* ≤ 0.05), as assessed using Student's *t*-test (a). Representative CYP1A1 immunoblot (24 hrs after biotin treatment). The intensities of the bands were normalized to GAPDH. The values are expressed as fold changes relative to the control group (dashed line). The (+) band corresponds to liver microsomes that were prepared from rats that had been treated with BaP (10 mg/kg), bands 2–5 are from control rats, and bands 6–9 correspond to biotin-treated rats (2 mg/kg). The molecular weights of the bands are indicated on the left: 57 kDa for CYP1A1 and 35 kDa for GAPDH. Four animals were used per group, and the data represent the results of two independent experiments (b).

**Figure 2:**
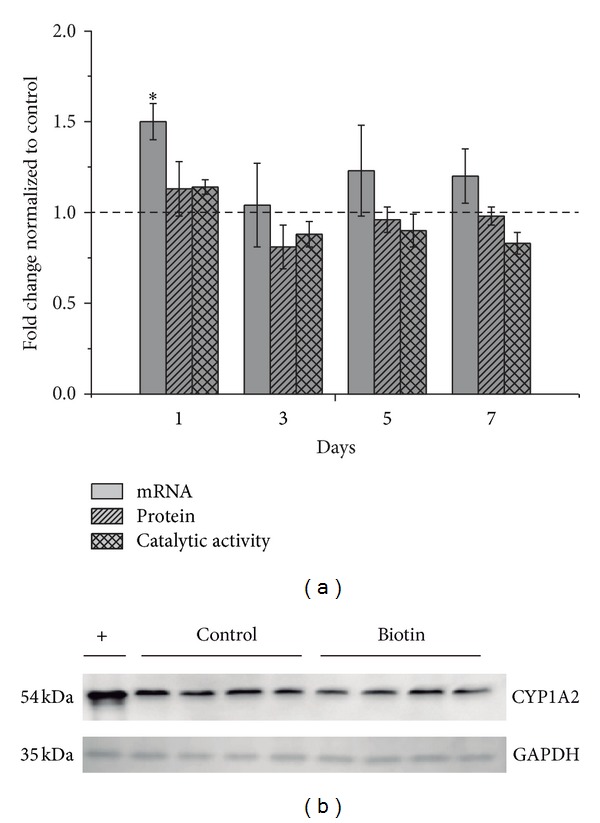
Relative CYP1A2 mRNA, protein, and catalytic activity levels after 1, 3, 5, and 7 days of biotin treatment (2 mg/kg, i.p.). The data are expressed as fold changes relative to the control group (dashed line). The data represent the means ± SEM of 4 rats per group from two independent experiments. *Significantly different from the controls (*P* ≤ 0.05), as assessed using Student's *t*-test (a). Representative CYP1A2 immunoblot (24 hrs after biotin treatment). The intensities of the bands were normalized to GAPDH. The values are expressed as fold changes relative to the control group (dashed line). The (+) band corresponds to liver microsomes that were prepared from rats that had been treated with BaP (10 mg/kg), bands 2–5 are from control rats, and bands 6–9 correspond to biotin-treated rats (2 mg/kg). The molecular weights are indicated on the left: 54 kDa for CYP1A2 and 35 kDa for GAPDH. Four animals were used per group, and the data represent the results of two independent experiments (b).

To evaluate whether pharmacological concentrations of biotin can modulate CYP1A expression in *in vivo* studies,we examined mRNA levels in the livers of rats that had been treated with biotin (2 mg/kg) at different times after administration. The results demonstrated that biotin significantly increased CYP1A1 and CYP1A2 mRNA levels (by 2.23 ± 0.22 and 1.50 ± 0.10 folds, resp.) after 24 hrs of biotin treatment, as shown in Figures 1(a) and 2(a). It is interesting to note that the increase in CYP1A1 decreased after 3 days (0.24 ± 0.01 fold decrease) compared with the control. At 5 and 7 days after biotin treatment, CYP1A1 and CP1A2 began to return to control levels.

### 3.2. Analysis of CYP Protein Expression

CYP protein expression in the rat livers was evaluated using Western blot (Figures 1(b) and 2(b)). Densitometric analyses of the bands revealed that the protein concentrations were not affected by biotin treatment on different days (*P* ≤ 0.05).

### 3.3. Effects of Biotin on CYP Activities

The levels of CYP1A1 and CYP1A2 activities were slightly higher than those in the controls at day 1, but the differences were not statistically significant (*P* ≤ 0.05). No changes were noted after 3, 5, and 7 days of biotin treatment (Figures 1(a) and 2(a)). These results corresponded to the effects that were observed at the protein level. The same microsomal samples that were used for the immunoblots were used for the enzymatic activity determinations. 

### 3.4. Effects of Biotin on BaP-Induced CYP Activity

**Figure 3:**
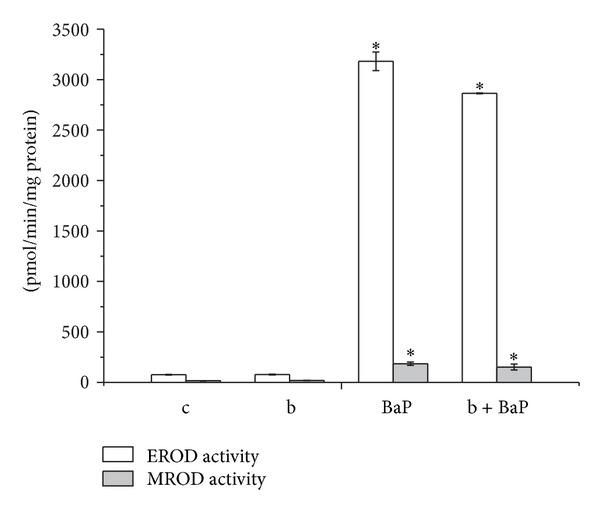
Effects of biotin treatment on EROD and MROD levels in the hepatic tissues of rats that were treated with a single i.p. dose of PBS 1X (control), biotin (2 mg/kg), BaP (10 mg/kg), or biotin + BaP (2 mg/kg of biotin plus 10 mg/kg of BaP). EROD activity: (pmol of resorufin formed/min/mg of protein); MROD activity: (pmol of resorufin formed/min/mg of protein). The results represent the means ± SEM. *n* = 5 rats per group. A *P* ≤ 0.05 was considered to be significantly different from the control group using Student's *t*-test.

Biotin was administered to rats in combination with BaP, a typical inducer of members of the CYP1A subfamily, to analyze whether the vitamin potentiated the capacity of BaP to increase CYP1A activity. The hepatic microsomal EROD and MROD activities were similar in animals that had been treated with either BaP alone or both biotin and BaP (Figure 3). 

### 3.5. Determination of the Possible Interactions between Biotin and the Catalytic Activities of CYP1A1 and CYP1A2

**Figure 4:**
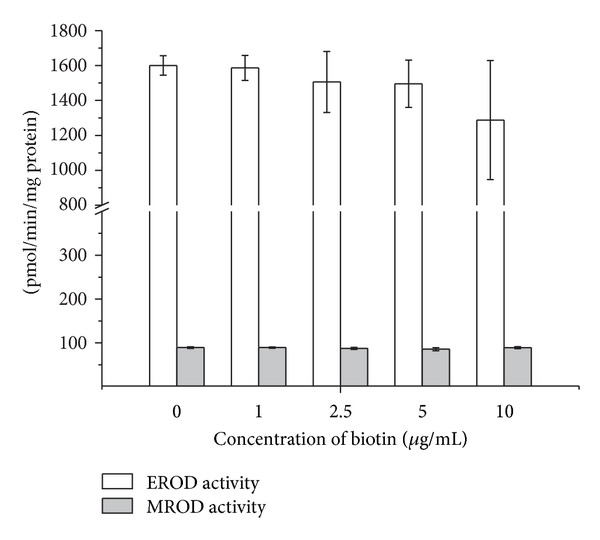
Effects of biotin treatment on EROD and MROD levels in the hepatic tissues of rats that were simultaneously treated with phenobarbital (60 mg/kg) and *β*-naphthoflavone (80 mg/kg). Increasing biotin concentrations were added to the reaction mixtures when measuring the enzymatic activity: (pmol of resorufin formed/min/mg of protein). The results represent the means ± SEM of two independent experiments that were conducted in triplicate. A *P* ≤ 0.05 was considered to be significantly different from the control group using Student's *t*-test.

To investigate whether biotin could interfere with the EROD and MROD activities, we used phenobarbital/*β*-naphthoflavone-induced rat liver microsomes. Different concentrations of biotin were added to the reaction mixtures with ER or MR. Biotin did not interfere with the activities of the CYP enzymes studied (Figure 4).

### 3.6. Ames Test

The mutagenic potencies of BaP resulting from its activation by S9 mixtures that were prepared from either the livers of biotin-treated rats or those prepared from the livers of control animals were very similar (Table 1). BaP had a mutagenic potency of 57.2 rev/*μ*g when activated by S9 mixtures that had been prepared from the livers of rats after 24 hrs of biotin treatment and a potency of 40.5 rev/*μ*g when activated by S9 mixtures that had been prepared from the livers of control rats. The mutagenic potencies of BaP that had been activated by hepatic S9 from rats that had been treated with biotin for 7 days and S9 that had been prepared from the control animals were 15.0 and 16.5 rev/*μ*g, respectively. In contrast, the mutagenic potencies of BaP that had been activated by S9 mixtures that had been prepared from rats that had been pretreated with a combination of known CYP inducers ranged from 37.4 to 62.3 rev/*μ*g.

### 3.7. Expression of miRNAs

**Figure 5:**
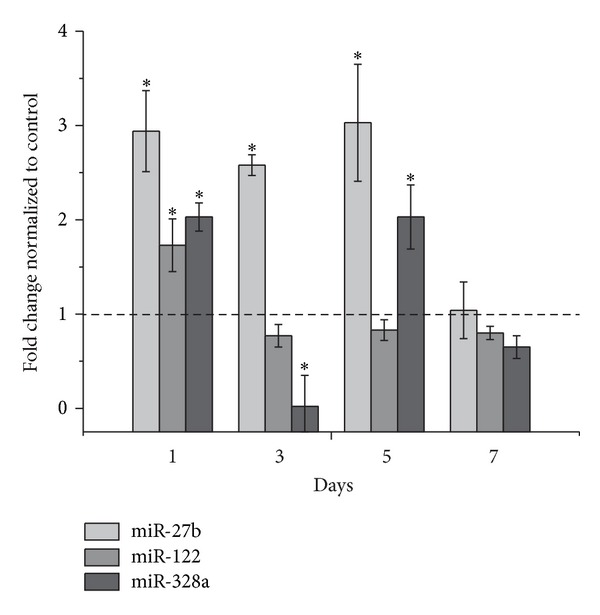
miR-27b, miR-122, and miR-328a levels after 1, 3, 5, and 7 days of biotin treatment (2 mg/kg, i.p.) in rat liver. The data are expressed as fold changes relative to the control (defined as 1, dashed line). The data represent the means ± SEM of 4 rats per group from two independent experiments. *Significantly different from the controls (*P* ≤ 0.05), as assessed using Student's *t*-test.

We also evaluated the levels of miR-27b, miR-122, and miR-328a in rat liver at different times after biotin treatment (2 mg/kg). The expression levels of the three miRNAs studied were increased after 24 hrs of biotin treatment. The levels of miR-27b remained increased after 3 and 5 days of biotin treatment, and the levels of the three miRNAs that were studied had returned to control levels after 7 days of treatment (Figure 5).

## 4. Discussion

Few studies have evaluated the toxicity of biotin [22–25]. Furthermore, studies that evaluated the toxicity of concentrations above normal levels need to be carefully readdressed given that the number of commercially available supplements that contain pharmacological amounts of the vitamin has increased [20]. In this work, we analyzed the effects of i.p. doses of 2 mg/kg of biotin on the expression and activity of members of the CYP1A subfamily that are involved in drug and xenobiotic metabolism. This dose of biotin is known to induce the expression of genes that are related to glucose metabolism [36].

Our results revealed that biotin administration modified CYP1A expression but did not alter the activity or protein concentrations of CYP1A (Figures 1 and 2). The finding that biotin administration did not affect CYP1A2 activity is of clinical interest because this enzyme is involved in the metabolism of numerous drugs, including clozapine, theophylline, tacrine, and verapamil, among others [37]. Therefore, alterations of the basal activity of CYP1A2 may cause clinically important drug-drug and food-drug interactions [38, 39]. These findings suggest that there is a low probability of drug-drug interactions resulting from the coadministration of biotin with drugs that are metabolized by CYP1A2. Additionally, CYP1A1 metabolizes polycyclic aromatic hydrocarbons (PAHs), which are ubiquitous compounds that are found in petroleum and emissions that are produced during the combustion of fossil fuels, in charred meat and fish that have been cooked directly over a fire, in tobacco smoke, and in several other products that are the result of the incomplete combustion of organic matter [40]. The metabolites that are formed from the interaction of CYP1A1 and PAHs are well known to generate DNA adducts leading to mutagenic events and chromosome aberrations that can contribute to the carcinogenic process [41]. Therefore, the lack of effect of biotin on CYP1A1 activity that was observed in this work supports the idea that exposure to biotin at pharmacologically relevant concentrations is harmless.

To further investigate the possible role of biotin in the modulation of members of the CYP1A subfamily, we explored the effects of biotin administration along with the known CYP inducer BaP, a typical substrate and inducer of these enzymes. No differences were detected in the activities of either CYP1A1 or CYP1A2 in hepatic microsomes that were obtained from rats that had been treated with the combination of biotin + BaP or BaP alone (Figure 3). Furthermore, biotin did not interfere with the activities of CYP1A1 or CYP1A2 when it was added to the reaction mixtures containing ER as a CYP1A1 substrate or MR as a CYP1A2 substrate, indicating that biotin does not act as an inhibitor for members of the CYP1A subfamily (Figure 4).

Additional evidence supporting the notion that biotin does not modulate the activity of the CYP enzymes studied was obtained using the Ames test [30]. This assay has been used to estimate the biological significance of CYP modulation, as the production of mutagenic metabolites from a known promutagen is dependent upon the CYP activity in the S9 mixture [42]. The S9 mixture that was prepared from rat livers that had been treated with biotin activated BaP at similar levels to that of the S9 mixture that had been prepared from the livers of control rats (Table 1). This result indicated that exposure to biotin did not alter the capacity of CYP1A1 to metabolize other substrates. Therefore, biotin may not influence CYP1A-mediated metabolism, further supporting its use as a therapeutic drug.

In contrast to the observed mRNA upregulation, CYP1A expression and enzyme activity were not significantly affected. These discrepancies may be explained by the fact that several studies have found that the abundance of selected proteins was not paralleled by the abundance of their mRNAs [7, 43]. In addition, pyruvate carboxylase apoenzyme expression is regulated by biotin at the posttranscriptional level, thereby influencing both protein synthesis and degradation [44]. Taken together, our observations suggest that biotin-based posttranscriptional effects may also influence CYP1A expression.

One possible explanation for our results could be posttranscriptional regulation that is mediated by the presence of miRNAs, which are small noncoding RNAs that regulate transcriptional and posttranscriptional gene expression [45]. In this process, not all of the mRNAs are translated immediately. Some of the mRNAs are maintained in a translationally repressed state and may be transported to a specific cytoplasmic location where translation is activated [46]. Recent studies have provided evidence that mRNAs that are silenced by miRNAs are localized in P-bodies for storage or degradation [47]. miR-27b, miR-122, and miR328a have been recently reported to be associated with the regulation of CYP enzymes [48–50]. Using in silico tools, such as BLAST and miRBASE, we identified target sequences for these miRNAs in the CYP1A subfamily. Our results (Figure 5) support the idea that the upregulation of these miRNAs by biotin could be implicated in the posttranscriptional regulation of CYP1A, although we cannot rule out that other posttranscriptional mechanisms may participate, given that biotin influences both protein synthesis and degradation [44]. Therefore, we propose that the observed changes to the CYP1A subfamily at the posttranscriptional level may have been directly produced by biotin or derived from a pleiotropic response that was triggered by biotin in the cell and that these changes cause an increase in small regulatory RNAs, such as miRNAs, which silence the subsequent translation of CYP1 mRNA. This hypothesis is currently being tested in experiments that are underway in our laboratory.

In conclusion, biotin administration affected the mRNA expression levels of CYP1A1 and CYP1A2. CYP1 mRNA induction had no effects at the protein or enzyme activity levels, suggesting that posttranscriptional regulation had occurred. The effects of biotin on CYP1A activity were confirmed using several different strategies. These results indicate that biotin does not interfere with CYP1A activity and suggest that biotin administration does not influence CYP1A1- and CYP1A2-mediated drug metabolism.

## Figures and Tables

**Table 1:** Evaluation of the mutagenicity of the S9 fraction that was obtained from rats that were treated with biotin in the Ames test.

Day	Chemical	Dose (*μ*g/plate)	Number of His^+^ revertants/plate^(a)^		
			B−^(b)^	B+^(c)^	Positive control^(d)^
1	B[a]P	0	23 ± 5	18 ± 1	28 ± 6
		1	55 ± 17	55 ± 8	59 ± 6
		5	229 ± 104	210 ± 59	433 ± 143
		10	215 ± 67	220 ± 61	557 ± 86
		20	304 ± 49	281 ± 30	913 ± 144
		Induced revertants/*μ*g^(e)^	40.5	52.7	62.3*

7	B[a]P	0	49 ± 8	62 ± 10	66 ± 7
		1	68 ± 7	66 ± 8	81 ± 27
		5	132 ± 7	137 ± 20	300 ± 29
		10	114 ± 17	128 ± 8	436 ± 99
		20	148 ± 31	159 ± 15	773 ± 127
		Induced revertants/*μ*g^(e)^	16.5	15.0	37.4*

^(a)^The mean number of revertant colonies identified in three replicates in two independent experiments.

^(b)^The liver S9 fraction from control rats.

^(c)^The liver S9 fraction from biotin-treated rats (2 mg/kg).

^(d)^The liver S9 fraction from phenobarbital-treated rats (60 mg/kg). *β*-naphthoflavone (80 mg/kg) was used as a positive control.

^(e)^The slope at the origin was calculated using the SALANAL software program.

*Significantly different from B− and B+ (*P* ≤ 0.05), as assessed using Student's *t*-test.
